# A procedure for removal of cyanuric acid in swimming pools using a cell-free thermostable cyanuric acid hydrolase

**DOI:** 10.1093/jimb/kuab084

**Published:** 2021-11-12

**Authors:** Feng Guo, Joseph C McAuliffe, Cristina Bongiorni, Jacob A Latone, Mike J Pepsin, Marina S Chow, Raj S Dhaliwal, Katherine M Hoffmann, Bill T Brazil, Meng H Heng, Serina L Robinson, Lawrence P Wackett, Gregory M Whited

**Affiliations:** Nutrition & Biosciences, International Flavors and Fragrances Inc., Palo Alto, 94304 CA, USA; Nutrition & Biosciences, International Flavors and Fragrances Inc., Palo Alto, 94304 CA, USA; Nutrition & Biosciences, International Flavors and Fragrances Inc., Palo Alto, 94304 CA, USA; Nutrition & Biosciences, International Flavors and Fragrances Inc., Palo Alto, 94304 CA, USA; Nutrition & Biosciences, International Flavors and Fragrances Inc., Palo Alto, 94304 CA, USA; Nutrition & Biosciences, International Flavors and Fragrances Inc., Palo Alto, 94304 CA, USA; Nutrition & Biosciences, International Flavors and Fragrances Inc., Palo Alto, 94304 CA, USA; Nutrition & Biosciences, International Flavors and Fragrances Inc., Palo Alto, 94304 CA, USA; Nutrition & Biosciences, International Flavors and Fragrances Inc., Palo Alto, 94304 CA, USA; Nutrition & Biosciences, International Flavors and Fragrances Inc., Palo Alto, 94304 CA, USA; Department of Environmental Microbiology, Eawag: Swiss Federal Institute for Aquatic Science and Technology, Überlandstrasse 133, CH-8600 Dübendorf, Switzerland; Department of Biochemistry, Molecular Biology and Biophysics, University of Minnesota, Minneapolis, 55455 MN, USA; Nutrition & Biosciences, International Flavors and Fragrances Inc., Palo Alto, 94304 CA, USA

**Keywords:** Cyanuric acid hydrolase, Biuret, Hypochlorite, Water treatment, *Pseudolabrys*

## Abstract

Cyanuric acid (CYA) is used commercially for maintaining active chlorine to inactivate microbial and viral pathogens in swimming pools and hot tubs. Repeated CYA addition can cause a lack of available chlorine and adequate disinfection. Acceptable CYA levels can potentially be restored via cyanuric acid hydrolases (CAH), enzymes that hydrolyze CYA to biuret under mild conditions. Here we describe a previously unknown CAH enzyme from *Pseudolabrys* sp. Root1462 (CAH-PR), mined from public databases by bioinformatic analysis of potential CAH genes, which we show to be suitable in a cell-free form for industrial applications based upon favorable enzymatic and physical properties, combined with high-yield expression in aerobic cell culture. The kinetic parameters and modeled structure were similar to known CAH enzymes, but the new enzyme displayed a surprising thermal and storage stability. The new CAH enzyme was applied, following addition of inexpensive sodium sulfite, to hydrolyze CYA to biuret. At the desired endpoint, hypochlorite addition inactivated remaining enzyme and oxidized biuret to primarily dinitrogen and carbon dioxide gases. The mechanism of biuret oxidation with hypochlorite under conditions relevant to recreational pools is described.

## Introduction

Hypochlorite is a widely used antimicrobial agent effective for mitigating the spread of pathogens and microorganisms, including bacteria, fungi, viruses, and parasites in water (Brady et al., 2002; Gardiner, 1973). Chlorine is often introduced into the pool water in the form of hypochlorous acid (HClO) or as a salt (NaClO). In aqueous solution, the hypochlorite ion (ClO^−^) exists in equilibrium with hypochlorous acid, chloride (Cl^−^), and molecular chlorine (Cl_2_) (see figures in the Supplementary Material) depending on pH, temperature, and concentration. In order to prevent the rapid photodegradation of hypochlorite (ClO^−^) and to maintain an effective hypochlorite concentration, the chlorine stabilizer cyanuric acid (CYA) is introduced into the system, thereby significantly slowing the chlorine/hypochlorite degradation process (see figures in the Supplementary Material). Trichloroisocyanuric acid and dichloroisocyanuric acid are typical chlorinated CYA stabilizers used to extend the duration of active chlorine “disinfection” in the pool industry.

Addition of CYA to the pool water slows the hypochlorite degradation process, but active chlorine still degrades or is consumed over time and must be replenished continuously throughout the operating period. Since the most common and convenient means of introducing and replenishing chlorine in the water is in the form of CYA, the level of CYA rises with each additional cycle of chlorine replenishment. This eventually results in the overstabilization of chlorine, with concomitant loss of the disinfection properties in the water. For example, overstabilization of chlorine, also referred to as “chlorine lock,” generally occurs when the concentration of CYA reaches over 100 ppm (∼0.77 mM), and its presence in this range signifies that the water may no longer have sufficient levels of free chlorine for effective disinfection.

There are several methods for removal of excess CYA from aqueous solutions; for example, adding melamine results in precipitation of melamine cyanurate, which is then removed by filtration (Somesla, 2013). Another method employs immobilized whole cells expressing an endogenous cyanuric acid hydrolase (CAH) (Radian et al., 2015; Yeom et al., 2015) or uses isolated CAH (Teichberg, 2008) (see figures in the Supplementary Material). However, the current state of the art for removing high concentrations of CYA (e.g. >50 ppm) continues to be partial or complete draining of the pool and subsequent replacement with fresh water.

CAHs are members of a relatively small protein family that also includes barbiturases and several proteins of yet unknown function (Seffernick et al., 2012). They all share a unique fold consisting of three domains that circumscribe the active site (Cho et al., 2014; Peat et al., 2013; Shi et al., 2019). The active site is defined by each domain contributing a triad of arginine, lysine, and serine, which together assemble a threefold symmetrical binding site for the threefold symmetric CYA substrate. This contributes to the enzyme's extraordinarily high degree of substrate selectivity. Several dozen compounds have been tested, but only CYA has been found to be reactive with CAHs (Fruchey et al., 2003). While the introduction of whole cells to the pool water is less efficient and the entire treatment may take up to 2 weeks, application of cell-free CAH to the pool is straightforward and more efficient. This strategy has been investigated and reported by several groups previously (Seffernick et al., 2012; Teichberg, 2008; Wackett et al., 2016), and several CAH homologs from different species have been studied to identify suitable enzyme candidates. Despite these efforts, no enzyme products are currently available for industrial application. The main reasons for this include the large volume of a typical residential pool, the amount of CAH required, and potential denaturation of CAH by residual free chlorine. Engineered *Moorella thermoacetica* CAH with the C46A mutation (Wackett et al., 2016) was shown to have improved hypochlorite resistance, but is still not sufficient for swimming pool applications with respect to hypochlorite sensitivity, enzyme production ability, and thermal stability.

To investigate and address this issue, we tested and screened putative CAH enzymes from a previous study (Aukema et al., 2020) and selected candidates based upon enzymatic performance, fermentation productivity, and thermostability. As a result, a previously unknown but well-performing CAH enzyme from *Pseudolabrys* sp. Root1462 (CAH-PR) was selected for CYA removal using active enzyme. In addition, we demonstrated that neutralization of residual hypochlorite by reducing agents (sodium sulfite or sodium thiosulfate), which has been well established in the pool industry, is critical for enzyme performance in pool water (see figures in the Supplementary Material). For the first time, we developed and reported a practical and economical method to chemically reduce residual hypochlorite before CAH treatment and conversion of CYA to biuret. Subsequent oxidation of biuret to gaseous by-products was achieved through reintroduction of hypochlorite into the pool, and inactivated the remaining CAH simultaneously. Overall, our study demonstrates a sustainable means to mitigate CYA accumulation in swimming pools over time with a substantial reduction in the need for freshwater dilution.

## Materials and Methods

### Expression of CAH Genes in *Bacillus subtilis*

Codon-optimized CAH genes were integrated into the *aprE* locus on the *B. subtilis* genome and constructed using standard molecular biology techniques as described previously (Bongiorni & Kaer, 2018). One microgram of the PCR product was mixed with 200 μl of competent *B. subtilis* cells expressing a *comK* gene (0.3% xylose induced) and comprising deletion of *alrA*, followed by recovery at 37°C for 1 hr. Single colonies were selected after incubation of the transformant on an LB plate at 37°C overnight. The genomic region containing the gene of interest was confirmed by Sanger sequencing. To examine the expression of CAH proteins, positive colonies were grown in Fernbach flasks in LB medium at 30°C for 24 hr. Cells were harvested and lysed by 0.1 mg/ml of lysozyme at 37°C for 1 hr before mixing with 2× SDS sample buffer and boiled for 10 min, followed by sodium dodecyl sulphate - polyacrylamide gel electrophoresis (SDS-PAGE) analysis (data not shown). Cells containing protein bands consistent with CAH expression were taken forward for the following work.

### Production, Recovery, and Granulation of CAH Proteins

*Bacillus subtilis* host cells showing positive expression of the expected CAH homologs were grown aerobically in a rich medium with glucose in 500 ml in Fernbach flasks as described earlier. After the specific protein production phase, the culture was harvested for protein recovery. Following lysozyme lysis, standard purification steps were implemented to isolate and purify the CAH proteins, which included filtration, the use of a polycationic polymer to aid debris separation, and concentration by ultrafiltration. Two previously untested CAH candidates, from *Bradyrhizobium diazoefficiens* (CAH-BD) and CAH-PR, were further produced via 2-l, glucose fed-batch stirred tank reactors. The CAH proteins were recovered and purified as described previously.

A prototype dry granule formulation of CAH was prepared under simulated real-world application conditions for swimming pool testing via fluid bed coating (Frey, 2014). Sodium sulfate cores, 200–350 μm in diameter, were coated with a concentrated solution of CAH-PR or CAH-BD comprising 100 g/l of protein and 15% of polyvinyl alcohol (PVA) in a fluid bed coater at less than 1.0% humidity. To these granules, a second coating of sodium sulfate was added at less than 1.0% humidity, followed by a third coat of 5% talc, 5% PVA, and 1.5% Neodol, and the final granule was dried at 45°C for 6 hr. The intention of this particular formulation was to use inorganic salts to minimize the amount of organic material that would be added to the swimming pool via the enzyme granules.

### Preparation and Assay of Enzymes

The performance of CAH enzymes in this study was measured by the modified melamine cyanurate precipitation assay (Downes et al., 1984). The assays were conducted in 96-well Costar 3628 microtiter plates (Corning, Glendale, AZ). Measurement of CYA consumption was not performed under standard substrate saturating conditions. Rather, reactions were set up to be more in line with concentrations and conditions found in swimming pool applications. CAH performance is observed and calculated by the change of CYA concentration over time in the different application tests versus the amount of CAH used. All assay data in this study were plotted and graphed in SigmaPlot (Systat Software, Palo Alto, CA).

Purified CAH was prepared as a 100× stock solution in buffer containing 0.1 M NaCl and 1× phosphate-buffered saline (PBS) (78.4 mM Na_2_HPO_4_, 21.6 mM NaH_2_PO_4_, 154.0 mM NaCl, pH 7.33), and 5 μl of enzyme was then mixed with 500 μl of CYA (200 ppm). After incubating at experimental conditions (30 min to 16 hr), 100 μl of each reaction was mixed with 100 μl of 2.5 mg/ml melamine and allowed to stand for 3 min as a white precipitate was formed, which was then measured by a SpectraMax M2e (San Jose, CA) at 600 nm. A linear curve of CYA standard versus optical density (OD) at 600 nm was plotted and used to determine the remaining CYA concentration for the samples, and the consumed CYA was calculated by subtracting the value from the initial CYA concentration. A standard curve of CYA was performed for every plate and every assay as a number of variables, such as temperature and other components in the reaction, can influence melamine cyanurate precipitation and the subsequent OD reading.

### Testing Hypochlorite Sensitivity of Enzymes

To test the sensitivity of CAH to hypochlorite, the reaction was carried out in simulated swimming pool water with up to 5.72 ppm hypochlorite (ClO^−^) (∼2× the ideal chlorine concentration in chlorinated pool water), 1 mM sodium bicarbonate (NaHCO_3_, pH 7.33), and 200 ppm CYA. Free chlorine and total chlorine were quantitatively determined by a modified DPD (*N*-diethyl-*p*-phenylenediamine) assay with a 96-well plate using a Taylor K-1001 kit (Hot Tub Warehouse). Each test sample (200 μl) and the hypochlorite (ClO^−^) standard solution (0–4 ppm) were transferred to a Corning 3641 96-well plate and mixed with 8 μl of DPD reagent #1 (buffer) and 8 μl of DPD reagent #2 (DPD) to determine free chlorine at 530 nm, while total chlorine was then measured by adding 8 μl of DPD reagent #3 (potassium iodide) to the same sample for A530 measurement thereafter. (Note: The measured chlorine level may be different with different chlorine standards, which was ∼8 ppm with 5.72 ppm hypochlorite in our case throughout this study.)

### Determining Relative Thermostability of CAH Homologs

Six CAH homologs were evaluated with respect to thermostability in conditions important to the intended application of pool remediation. In this experiment, CAH proteins were subjected to a 30 min aqueous incubation at indicated temperatures. Each CAH enzyme was diluted to ∼1 mg CAH extract/ml in 1× PBS (pH 7.33) and 0.1 M NaCl and transferred to a PCR tube, followed by 30 min incubation at 25, 35, 45, 55, 65, and 75°C. Following the temperature incubation, all samples were diluted to 0.5 mg CAH extract per milliliter with the same buffer and 2.5 μl was mixed with 250 μl of 200 ppm CYA and incubated at room temperature for 30 min to determine the residual enzyme activity by quantifying the residual undigested CYA using melamine cyanurate precipitation assay. Each CAH enzyme's residual activity at various temperatures was normalized to the sample at 25°C and is shown in Fig. 7.

To further investigate the stability of CAH as a product, we prepared prototype granules of both CAH-PR and CAH-BD on a small scale (as described earlier) for an aging test. To accelerate the aging of granules, the test was performed with stress conditions, such as high temperature and high humidity, while the same aging progress may take much longer under more mild conditions. Granules, 0.5–1.0 g, of CAH-PR or CAH-BD (18.5% payload) were weighed into test tubes and incubated at the following conditions with duplicates: (1) room temperature, (2) 40°C with 75% humidity; (3) 50°C with 65% humidity, and (4) 50°C (dry condition). At time points of 0, 1, 2, 4, and 8 weeks, samples were dissolved in buffer with 0.1 M NaCl and 1× PBS (pH 7.33) to a final concentration of 10 mg/ml CAH. Each sample was diluted to a final concentration of 0.4 mg/ml with the same buffer for enzymatic activity test, where 2.5 μl of each 0.4 mg/ml CAH was mixed with 250 μl of 200 ppm CYA and incubated at room temperature for 35 min before being measured by melamine cyanurate precipitation assay.

### Nuclear Magnetic Resonance Materials and Instrumentation

^13^C_3_/^15^N_3_-CYA (90%+; ^13^C_3_ 99%, ^15^N_3_ 98%+) was purchased as a 1 mg/ml (7.4 mM) solution in water (Cambridge Isotope Laboratories, Tewksbury, MA). An aliquot (650 μl) of this solution was mixed with D_2_O (50 μl) containing an internal standard (sodium trimethylsilylpropanesulfonate [DSS], 0.75% wt/wt) and the ^13^C nuclear magnetic resonance (NMR) spectrum obtained at 125.6 MHz on a Varian VNMRS 500 MHz NMR system running VNMRJ 4.2 software and outfitted with a tunable, broadband 5 mm probe. A standard carbon pulse sequence was applied with 128 transients at 298 K, an acquisition time of 1.3 s, and a relaxation delay of 1 s.

For ^15^N NMR, an aliquot (650 μl) of ^13^C_3_/^15^N_3_-CYA solution (1 mg/ml) was mixed with D_2_O (50 μl) containing an internal standard (DSS, 0.75% wt/wt) and the ^15^N NMR spectrum obtained at 50.6 MHz on a Varian VNMRS 500 MHz NMR system running VNMRJ 4.2 software and outfitted with a tunable, broadband 5 mm probe. A standard ^15^N pulse sequence was applied with 484 transients at 298 K, an acquisition time of 3.2 s, and a relaxation delay of 100 s.

### Gas Chromatography/Mass Spectrometry Analysis of ^15^N-Urea and ^15^N-Biuret to Determine the Mechanism of Hypochlorite Oxidation

The mechanism by which dinitrogen (N_2_) was formed upon oxidation of both urea and biuret with hypochlorite was studied using a 1:1 mixture of ^14^N- and ^15^N-labeled standards, followed by gas chromatography/mass spectrometry (GC/MS) analysis of the resulting nitrogen isotopomers present in both N_2_ and nitrous oxide (N_2_O).

Unlabeled urea (^14^N_2_, 99.63% isotopic purity; 500 μl of 20 mM, 10 μmol) and ^15^N_2_-urea (99%+ isotopic purity, Cambridge Isotope Laboratories, Andover, MA; 500 μl of 20 mM, 10 μmol) were added to each of six 20 ml glass headspace vials (Agilent, Santa Clara, CA, USA). Sodium phosphate buffer (18 ml of 10 mM) at three different pH values (pH 5, 7, and 10) was added to the vials in duplicate for each pH value. Commercial sodium hypochlorite bleach (Clorox, Oakland, CA) (250 μl of ∼1 M, 250 μmol) was added to each vial. Water was then added dropwise to overfill each vial, followed by a headspace vial cap to exclude ambient air.

A second set of six headspace vials (duplicates at pH values of 5, 7, and 10) was prepared in the same manner as for urea, but with unlabeled biuret (^14^N_3_, 99.63% isotopic purity; 1 ml of 10 mM, 10 μmol) and ^15^N_3_ biuret (99%+ isotopic purity, Sigma Aldrich, Milwaukee, WI; 1 ml of 10 mM, 10 μmol) for a final biuret concentration of 1 mM (∼136 ppm).

After overnight reaction at room temperature, argon gas (∼4 ml) was injected into the inverted vials and allowed to pressure equalize by removal of the same volume of liquid. A metal foil seal was placed on the headspace vial cap to prevent any gas loss or admission of air. The vials could then be directly analyzed by headspace GC/MS without the need to transfer gas to a separate vial. The needle penetration depth of the autosampler was carefully adjusted such that only gas was withdrawn from each vial during GC/MS analysis.

Headspace GC/MS analysis of the vials was performed on an Agilent 6890 GC with 5973 mass selective detector (MSD) outfitted with a CTC CombiPAL autosampler operating in headspace mode. A 1 ml headspace syringe was used to withdraw 500 μl of gas and was injected into an inlet held at 150°C with a 50:1 split. An Agilent HP-PoraPLOT Q column (30 m × 0.32 mm × 20 μm) held at 40°C and eluted with helium flowing at 1.5 ml/min was used to separate N_2_/O_2_/argon (RT 1.84 min) from carbon dioxide (2.62 min) and nitrous oxide (2.88 min). The 5973 MSD was used in full scan mode from *m*/*z* 26 to 100. Raw data files acquired using Agilent Chemstation (version D.03.00.611) were exported in .cdf format and imported into Chromeleon 7.3 software (Thermo Fisher, San Jose, CA). Total ion chromatograms (TICs) were extracted for ions 28/29/30 (for N_2_) and 44/45/46 (for N_2_O). The integrated area for each ion was determined and the ratios of *m*/*z* 29–30 and *m*/*z* 44/45/46 determined. The background amount of *m*/*z* 29 was calculated (based on *m*/*z* 28) and removed from the total *m*/*z* 29 ion amount. Formulas 1 and 2 were used for converting the ratio of ions 29 and 30 to the extent of intramolecular N_2_ formation (expressed as percentage intramolecular reaction) for urea and biuret, respectively.

For urea:

(1) \begin{eqnarray*} \% \,{\rm{Intra}} = - 100\left( {x + 2} \right)/(x - 2),\,{\rm{where}}\,x = m/z29/30\,{\rm{ratio}} \end{eqnarray*}

For biuret:

(2) \begin{eqnarray*} \% \,{\rm{Intra}} = - 150\left( {x + 2} \right)/(x - 2),\,{\rm{where}}\,x = m/z\,29/30\,{\rm{ratio}} \end{eqnarray*}

## Results

### Identifying Proteins Comprising CAH Activity by Amino Acid Sequence Pattern

**Fig. 1.**
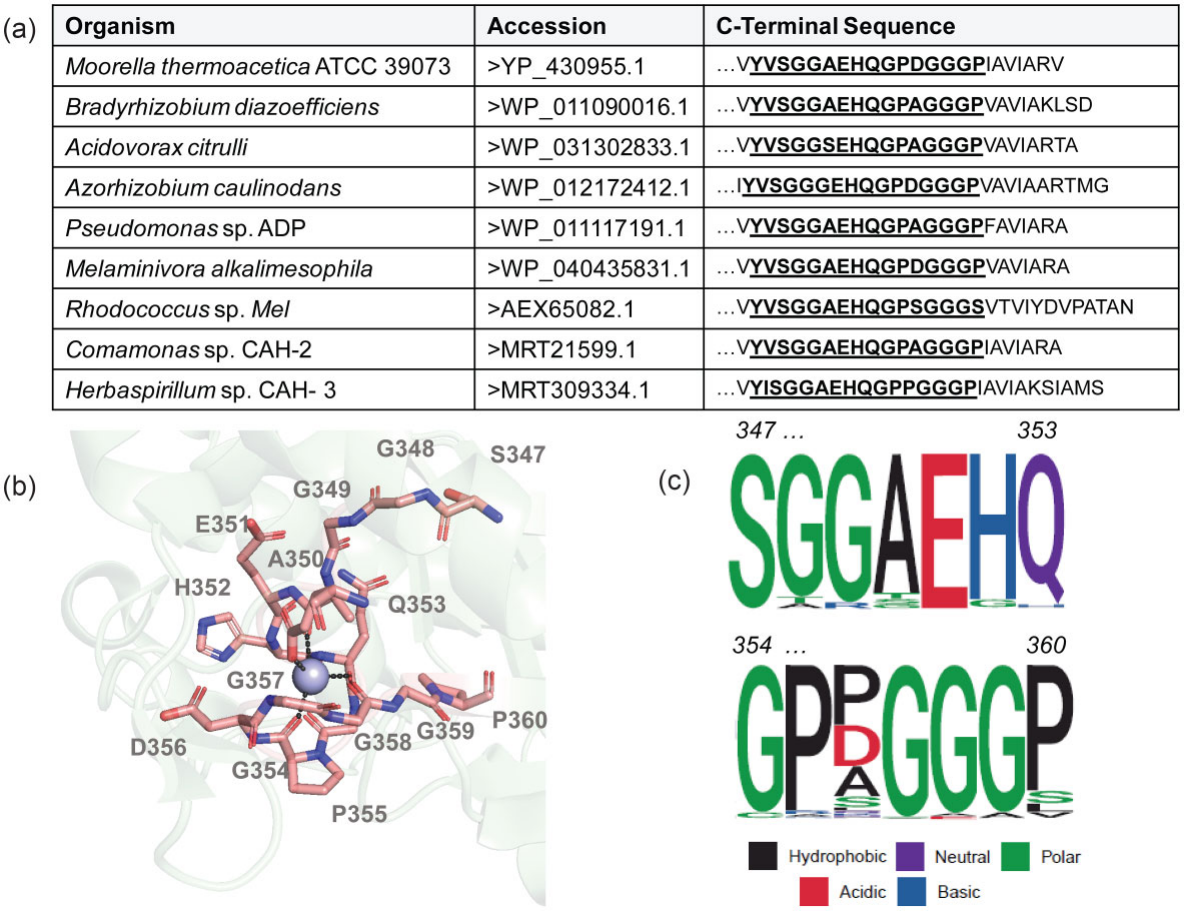
Bioinformatic analysis defines the highly conserved C-terminus metal binding domain as diagnostic sequence pattern for cyanuric acid hydrolase (CAH) identification. (a) Gold standard CAHs from different bacteria for which activity has been directly demonstrated and showing the specific sequences defining the sequence pattern region. (b) Mapping of sequences onto the X-ray structure of the CAH from *Moorella thermoacetica* (6BUM) in the C-terminal region that contains residues of the metal binding site. (c) Derived sequence pattern shown for all known and predicted CAHs. All of the sequences’ patterns are near the C-termini. The numbering differs slightly. The specific amino acid numbers shown are for the CAH from *M. thermoacetica* ATCC39073.

A limited number of CAHs have been purified to homogeneity and tested for their reactivity (Fig. 1a). CAHs in Fig. 1 are from taxonomically distinct bacteria in the Proteobacteria and Actinobacteria phyla and are members of a protein family that also includes enzymes known as barbiturases. One defining feature of CAHs relative to barbiturases is two conserved arginine residues aligning with R194 and R324 in the *Pseudomonas* sp. ADP sequence (Seffernick et al., 2012). Another defining CAH motif, S-G-G-X-E-X-Q-G-P-X-G-G-G-P (Fig. 1b, generated by PyMOL [Yuan et al., 2016]), was identified in this study through sequence alignment of >600 plausible CAHs (Aukema et al., 2020) (Fig. 1c, generated by the ggseqlogo package [Wagih, 2017]). The utility of S-G-G-X-E-X-Q-G-P-X-G-G-G-P as a sequence motif was further supported by characterization of new CAHs in this study and represents a further, and slightly more restrictive, methodology for CAH identification.

Subsequently, we examined six proteins from five different genera of bacteria that matched the pattern described in Fig. 1 for putative CAH proteins. The six proteins included three represented in Fig. 1, *M. thermoacetica* (WT and C46A) (accession ID: WP_011393610.1) (Shi et al., 2019), *B. diazoefficiens* (formerly *Bradyrhizobium japonicum* USDA110, accession ID: WP_011090016.1) (Seffernick et al., 2012)*, Acidovorax citrulli* (accession ID: WP_031302833.1) (Bera et al., 2017), and two putative CAH enzymes from *Pseudolabrys* sp. Root1462 (accession ID: WP_056911810.1) and *Bradyrhizobium* sp. WSM1253 (accession ID: WP_007596559.1). All were produced by growth and expression in a *B. subtilis* host strain (as described in the Materials and Methods) for evaluation of production and to test their sensitivities to hypochlorite under identical conditions.

### Performance of CAH Candidates in Simulated Swimming Pool Water Conditions

A typical range of free chlorine in swimming pools is ∼1–3 ppm. Because chlorine is an oxidant that can interact with various organic molecules, including proteins, chlorinated pool water can kill most pathogens and microbials, but this also is a significant challenge for enzyme treatment. Most enzymes may lose activity or biological function in the presence of hypochlorite. Therefore, a comprehensive evaluation of the sensitivity of CAH candidates to free chlorine is a key step for the development of enzymatic CYA treatment.

**Fig. 2.**
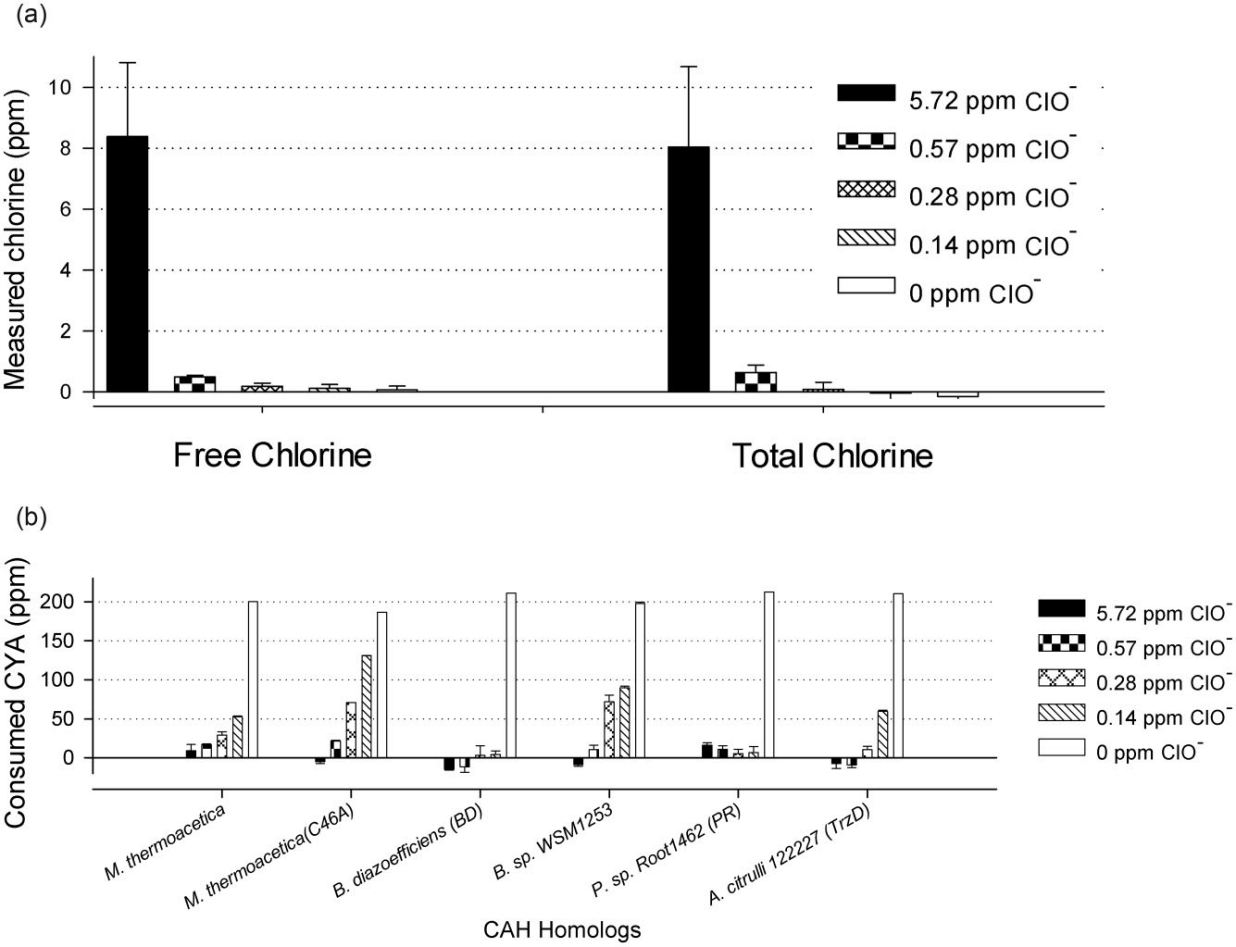
Cyanuric acid hydrolases (CAHs) were inactivated at hypochlorite level in pool condition (0.14–5.72 ppm). (a) Measured free and total chlorine level with 0–5.72 ppm of hypochlorite (ClO^−^) by DPD (*N*-diethyl-*p*-phenylenediamine) assay. Commercial bleach (8.25% of NaClO) was diluted to 0–5.72 ppm of hypochlorite in simulated pool water (1 mM NaHCO_3_, pH 7.33; 200 ppm cyanuric acid [CYA]). The actual free and total chlorine levels were quantitatively determined by DPD assay before testing CAH performance under these conditions. (b) Performance of CAH homologs from *Moorella thermoacetica* (WT and C46A) (WP_011393610.1), *Bradyrhizobium diazoefficiens* (WP_011090016.1), *Pseudolabrys* sp. Root1462 (WP_056911810.1), *Bradyrhizobium* sp. WSM1253 (WP_007596559.1), and *Acidovorax citrulli* 122227 (WP_031302833.1) at 0–5.72 ppm of hypochlorite in the presence of 1 mM NaHCO_3_, pH 7.33, and 200 ppm CYA. The *Y*-axis represents the amount of CYA (ppm) consumed by CAH enzymes at RT in 16 hr.

**Fig. 3.**
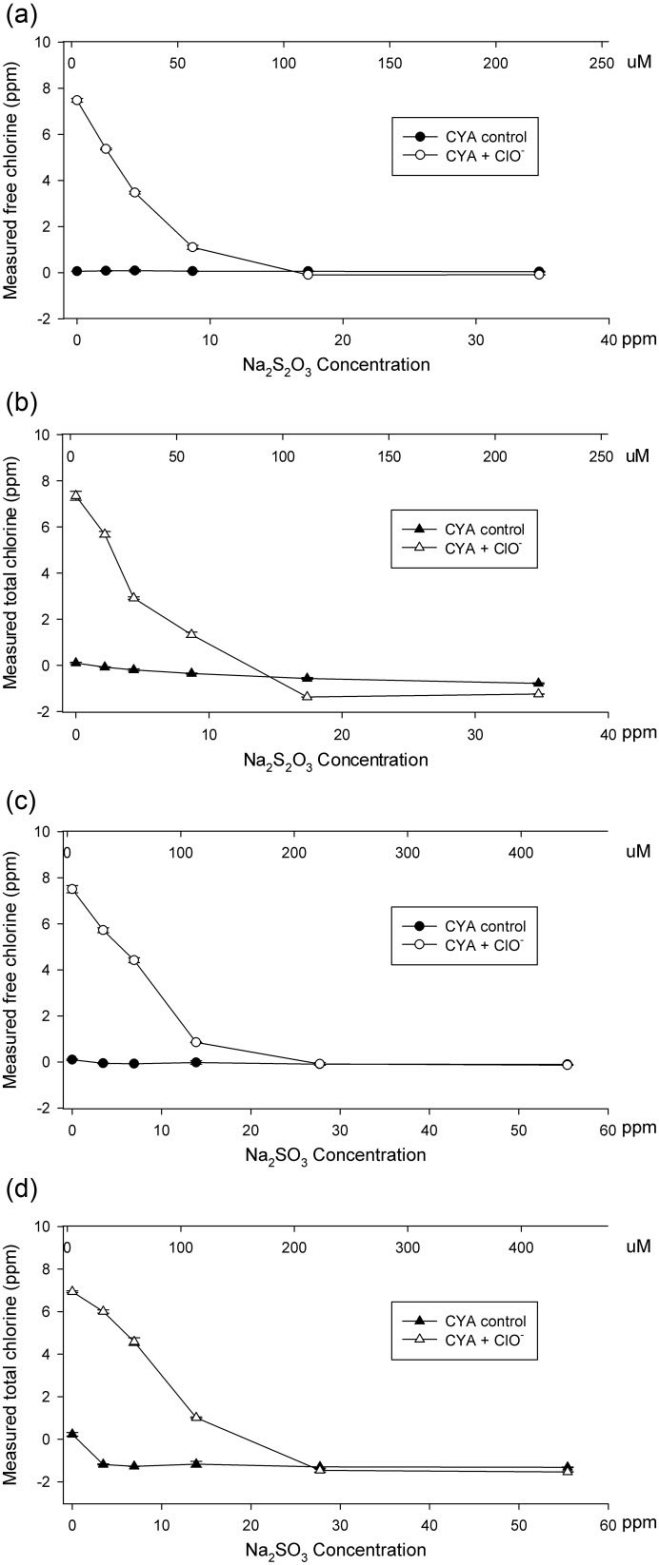
Determination of sodium thiosulfate and sodium sulfite amount for hypochlorite reduction in simulated pool water. (a,b) Titration of sodium thiosulfate against 5.72 ppm NaClO in simulated pool water. Free and total chlorine in simulated swimming pool water (1 mM NaHCO_3_, pH 7.33; 200 ppm cyanuric acid; 5.72 ppm NaClO) was monitored by DPD (*N*-diethyl-*p*-phenylenediamine) assay when titrated by 0–34.8 ppm sodium thiosulfate. (c,d) Titration of sodium sulfite against 5.72 ppm NaClO in simulated pool water. Free and total chlorine in simulated swimming pool water (same as (a,) was monitored by DPD assay when titrated by 0–55.4 ppm sodium sulfite.

As shown in Fig. 2a, free chlorine can be detected as low as 0.14 ppm by modified DPD assay with 200 ppm CYA. Each CAH enzyme (0.3–2 mg/l) was incubated with CYA (200 ppm) and hypochlorite (0–5.72 ppm) for 16 h at room temperature in simulated pool water, where all six CAH proteins were inactivated (Fig. 2b), even with as low as 0.14–0.57 ppm hypochlorite. This demonstrated that the presence of even trace amounts of hypochlorite in swimming pools will inactivate CAH enzymes, which prevents hydrolysis of CYA under this condition. Sodium sulfite and sodium thiosulfate are routinely used in the swimming pool industry as reductants to lower the concentration of active or free chlorine when they are inadvertently added to unwanted levels (Fig. S1[4–5]). To determine the amount of reductant for neutralization in this study, we titrated against 5.72 ppm hypochlorite in simulated pool water with a series of concentrations of sodium sulfite or sodium thiosulfate. As shown in Fig. 3, bleach samples (Clorox, Oakland, CA) were titrated with 0, 2.2, 4.3, 8.7, 17.4, and 34.8 ppm sodium thiosulfate (Na_2_S_2_O_3_), and 200 μl of each sample was taken to determine the free chlorine and total chlorine by the modified DPD assay (Figs 3a and b). Another set of samples was titrated with 0, 3.4, 6.9, 13.9, 27.7, and 55.4 ppm sodium sulfite (Na_2_SO_3_) to reduce hypochlorite (ClO^−^) (Figs 3c and d). As a result, 17.4ppm sodium thiosulfate (Na_2_S_2_O_3_) or 27.7 ppm sodium sulfite (Na_2_SO_3_) was enough to reduce 5.72 ppm hypochlorite (ClO^−^). These results demonstrate that a small amount of sodium thiosulfate or sodium sulfite can be applied to the pool water to neutralize hypochlorite and generate a neutral or reducing environment for mitigating CYA via CAH enzymes.

### CAH Enzymes Maintain Activity in Simulated Pool Water After Hypochlorite Neutralization

**Fig. 4.**
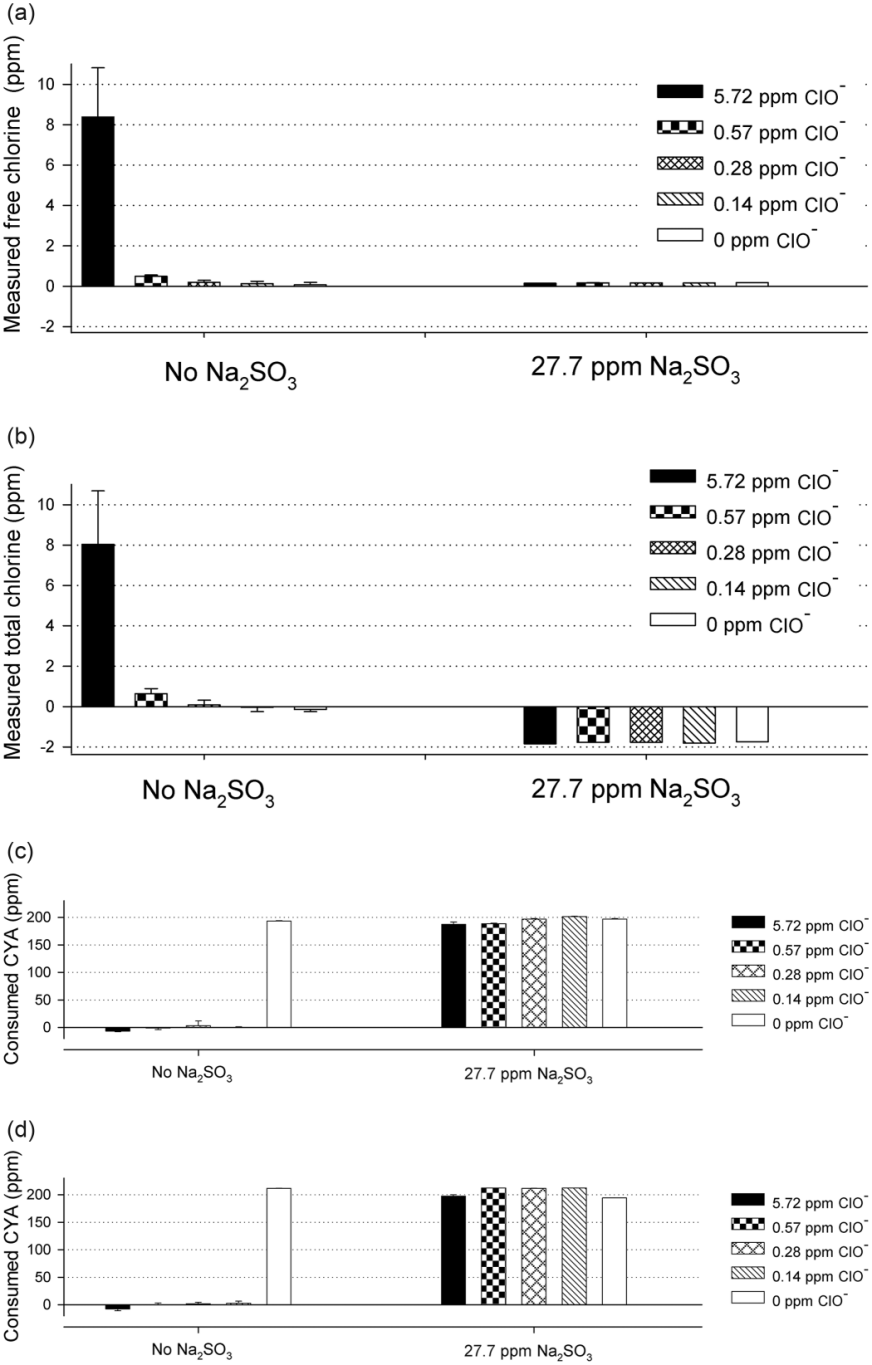
Both CAH-BD (*Bradyrhizobium diazoefficien*) and CAH-PR (*Pseudolabrys* sp. Root1462) retained the capability to digest cyanuric acid (CYA) after reducing hypochlorite by sodium sulfite in simulated pool water. (a,b) Free and total chlorine was reduced to at or below zero after addition of 27.7 ppm sodium sulfite in simulated pool water (1 mM NaHCO_3_, pH 7.33; 200 ppm CYA). (Note: the total chlorine level of deionized H_2_O is considered to be 0. The negative value of measured total chlorine level indicates the redox level is more reduced than deionized H_2_O.) Free and total chlorine levels with 0–5.72 ppm hypochlorite (NaClO) were determined by DPD (*N*-diethyl-*p*-phenylenediamine) assay, before (left panel) and after (right panel) addition of 27.7 ppm sodium sulfite. (c) Performance of CAH-BD to hydrolyze CYA before (left panel) and after (right panel) reducing hypochlorite by 27.7 ppm sodium sulfite in simulated pool water at room temperature (RT) for 7 hr. The *Y*-axis represents the amount of CYA (ppm) consumed by cyanuric acid hydrolase (CAH). (d) Performance of CAH-PR before (left panel) and after (right panel) reducing hypochlorite by 27.7 ppm sodium sulfite in simulated pool water at RT for 7 hr. The *Y*-axis represents the amount of CYA (ppm) consumed by CAH.

**Fig. 5.**
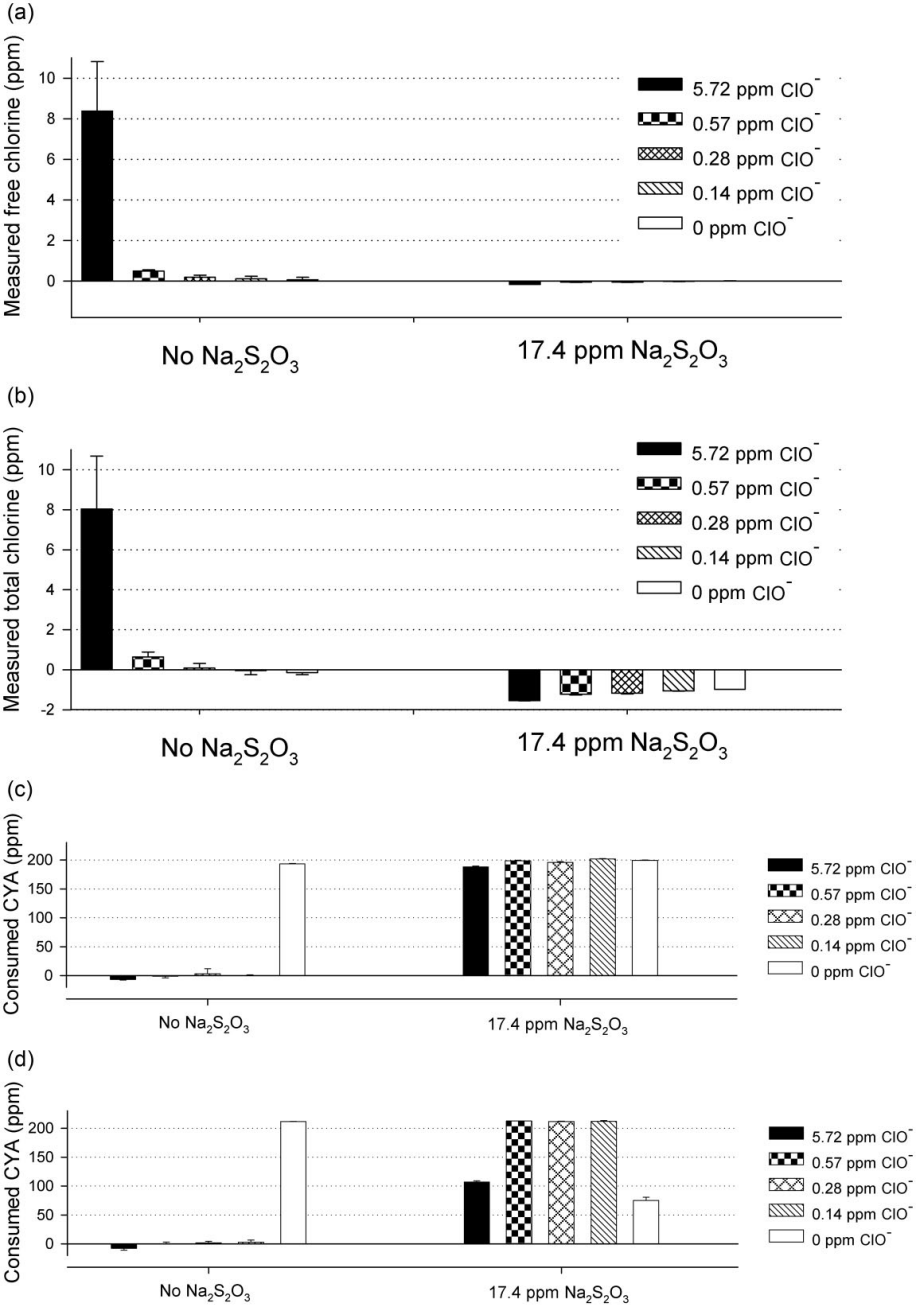
Both CAH-BD (*Bradyrhizobium diazoefficien*) and CAH-PR (*Pseudolabrys* sp. Root1462) retained the capability to digest cyanuric acid (CYA) after reducing hypochlorite by sodium thiosulfate in simulated pool water. (a,b) Free and total chlorine was reduced to or below zero after addition of 17.4 ppm sodium thiosulfate. (Note: the total chlorine level of deionized H_2_O is considered to be zero; the negative value of measured total chlorine level indicates the redox level is more reduced than deionized H_2_O.) Free and total chlorine levels with 0–5.72 ppm hypochlorite (NaClO) in simulated pool condition (1 mM NaHCO_3_, pH 7.33; 200 ppm cyanuric acid [CYA]) were measured by DPD (*N*-diethyl-*p*-phenylenediamine) assay, before (left panel) and after (right panel) addition of 17.4 ppm sodium thiosulfate. (c) Performance of CAH-BD before (left panel) and after (right panel) reducing hypochlorite by 17.4 ppm sodium thiosulfate in simulated pool water at room temperature (RT) for 7 hr. The *Y*-axis represents the amount of CYA (ppm) consumed by CAH. (d) Performance of CAH-PR before (left panel) and after (right panel) reducing hypochlorite by 17.4 ppm sodium thiosulfate in simulated pool water at RT for 7 hr. The *Y*-axis represents the amount of CYA (ppm) consumed by CAH.

To investigate whether CAH could maintain activity after neutralizing hypochlorite by reductants, CAH-BD and CAH-PR were tested for their performance before and after the neutralization with sodium sulfite (27.7 ppm Na_2_SO_3_; Fig. 4) or sodium thiosulfate (17.4 ppm Na_2_S_2_O_3_; Fig. 5). Free and total chlorine was measured with and without reductants by the modified DPD assay, where the reductants completely neutralized hypochlorite (right panel of Figs 4a and b and Figs 5a and b). After that, CAH-BD (0.4 mg/l) or CAH-PR (0.45 mg/l) was added to 1 ml of each sample and incubated for 7 h at room temperature. CAH performance was then determined by melamine cyanurate precipitation assay via residual CYA concentration. As shown in Figs 4c and d (left panel), both enzymes were inactivated in the presence of hypochlorite (0.14–5.72 ppm) but retained most of their activities when hypochlorite was neutralized by sodium sulfite (27.7 ppm) prior to the addition of CAH (Figs 4c and d, right panel). When sodium sulfite was overdosed for a low concentration of hypochlorite (0.14–0.57 ppm), no negative effect to either CAH-BD or CAH-PR was observed. Similar results were obtained with sodium thiosulfate (17.4 ppm) neutralization, as shown in Figs 5c and d. While overdosed sodium thiosulfate had no negative effect on CAH-BD performance under these conditions, CAH-PR only maintained partial activity after sodium thiosulfate neutralization with 5.72 ppm hypochlorite and was inhibited by sodium thiosulfate with 0 ppm hypochlorite.

To extend this observation and confirm that chemical reduction of hypochlorite is a generally useful procedure for other CAHs (not specific to CAH-PR and CAH-BD), all six CAH candidates were tested following the same protocol but at the high end of hypochlorite (5.72 ppm). When CAHs were added to the mock swimming pool water containing 5.72 ppm hypochlorite, all enzymes were inactivated. However, when these test conditions were treated with sodium sulfite (Na_2_SO_3_, 27.7 ppm) or sodium thiosulfate (Na_2_S_2_O_3_, 17.4 ppm) before the addition of CAHs, the enzyme performance was maintained at, or near, the positive (+) control level (data not shown).

### CYA Hydrolysis With Broad Range of Substrate Concentrations

**Fig. 6.**
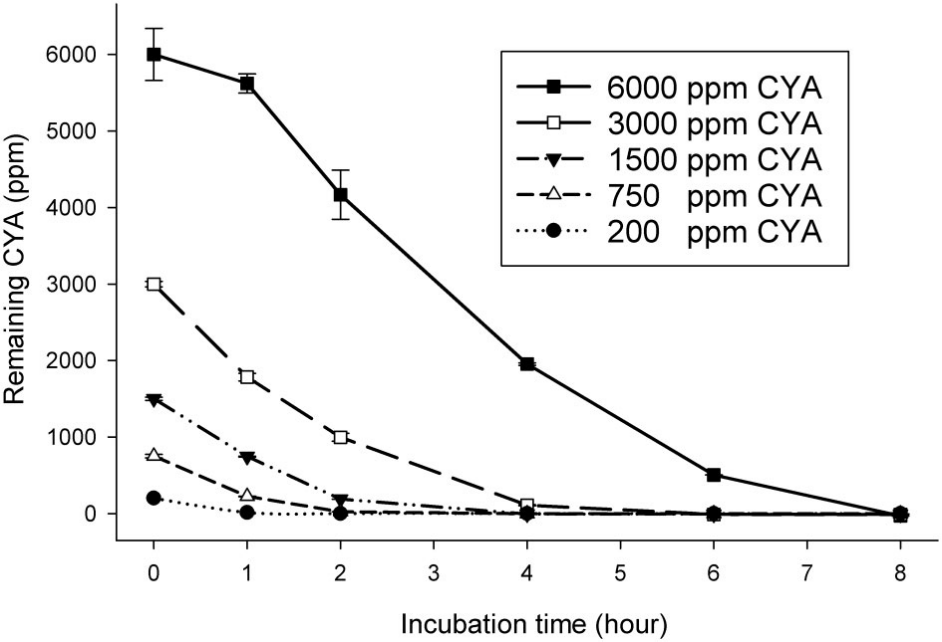
CAH-PR (*Pseudolabrys* sp. Root1462) hydrolyzed cyanuric acid (CYA) at concentrations ranging from 200 to 6,000 ppm. A series of concentrations of CYA at 200, 750, 1,500, 3,000, and 6,000 ppm was prepared in deionized water and incubated with 7.5 mg/l CAH-PR at room temperature for an 8-hr time course. Samples were taken at 0, 1, 2, 4, 6, and 8 hr and the reactions were terminated by addition of 5.72 ppm hypochlorite. The *Y*-axis represents the amount of CYA consumed by CAH-PR during incubation.

Although the experiments described herein generally use 100–200 ppm CYA, CAH-PR could hydrolyze various concentrations of CYA, ranging from 200 to 6,000 ppm. As shown in Fig. 6, CAH-PR (7.5 mg/l) was added to 1 ml of CYA at 6000, 3000, 1500, 750, and 200 ppm and incubated at room temperature. The reactions were terminated at the indicated times, by addition of 5.72 ppm hypochlorite, and frozen on dry ice. All the samples were thawed at the end of incubation and diluted to what would be 200 ppm CYA if there had been no reaction. The actual CYA concentration was determined using melamine cyanurate precipitation assay. CYA hydrolysis proceeded at a constant rate that was CAH dependent and is consistent with observations that the reaction is not product (biuret) inhibited. The loss of carbon dioxide (CO_2_) during the forward CAH hydrolysis reaction (see Figs S1[3] and S2A,B) makes the reaction effectively irreversible.

### Relative Thermostability of CAH Homologs

**Fig. 7.**
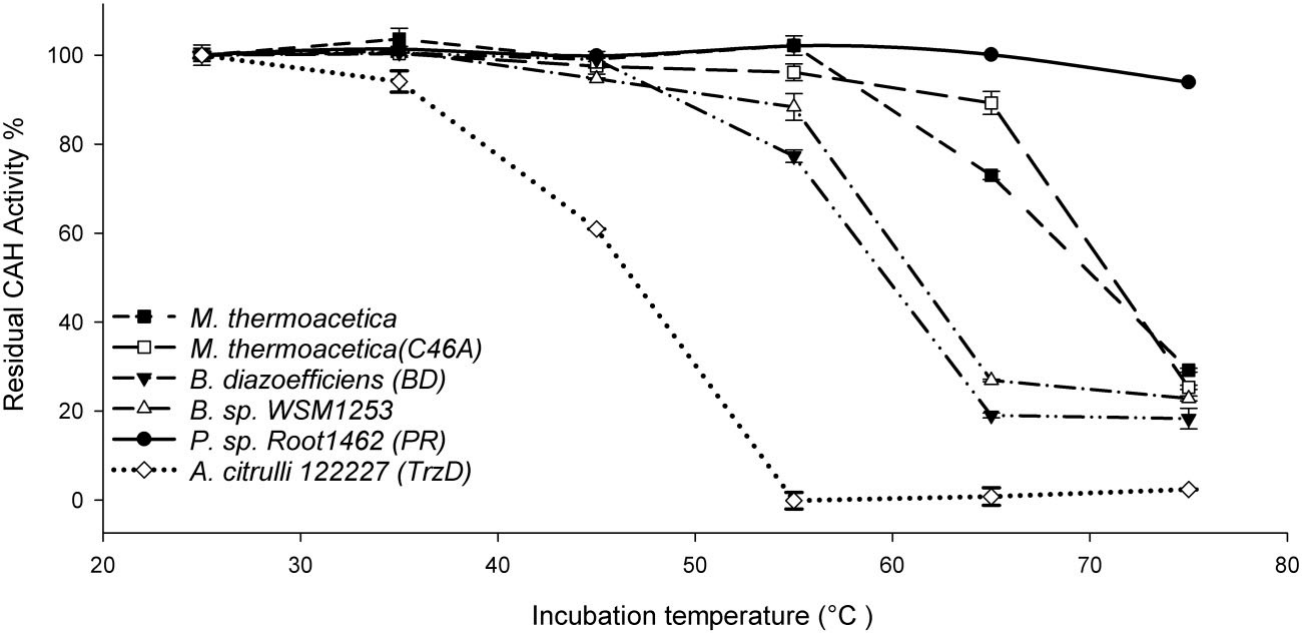
Thermostability test of six CAH homologs. Six CAH homologs from five different bacteria genera that matched the sequence pattern described in Fig.1 were expressed in *Bacillus subtilis* to compare the retained activity at different temperatures, including CAH from *Moorella thermoacetica* (WT and C46A), *Bradyrhizobium diazoefficiens, Acidovorax citrulli, Pseudolabrys* sp. Root1462, and *Bradyrhizobium* sp. WSM1253. After incubation at 25, 35, 45, 55, 65, and 75°C for 30 min, retained activity was determined by the consumed cyanuric acid amount at room temperature for 30 min and normalized to the value at 25°C for plotting.

**Fig. 8.**
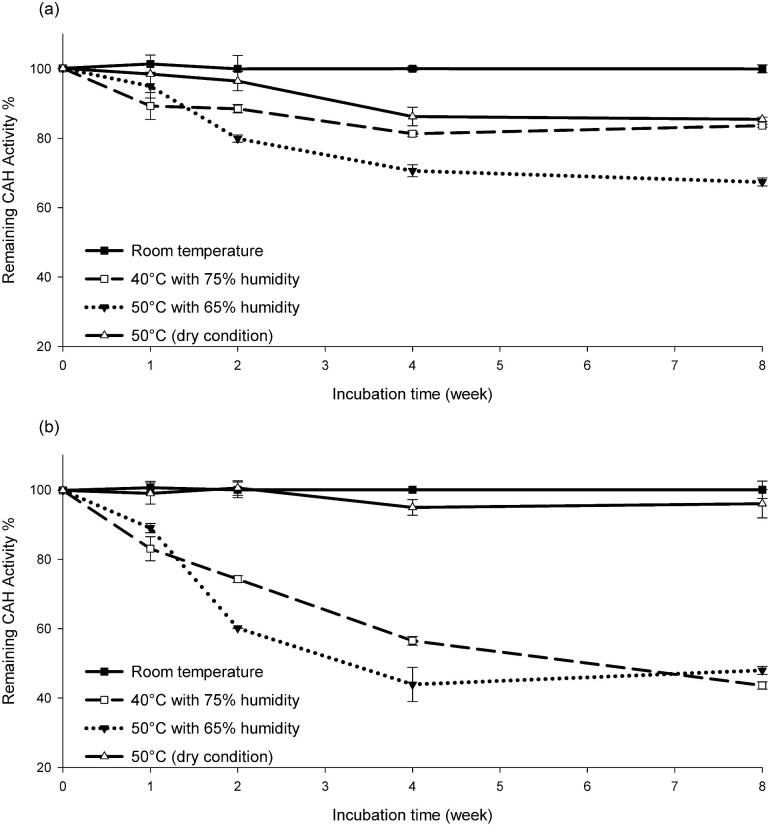
Eight-week aging test of CAH-PR (*Pseudolabrys* sp. Root1462) and CAH-BD (*Bradyrhizobium diazoefficien*) granules. (a) CAH-PR and (b) CAH-BD granules were incubated at the following conditions in duplicate: (1) room temperature (solid line, black), (2) 40°C with 75% humidity (dashed line, gray), (3) 50°C with 65% humidity (dotted line, gray), (4) 50°C (dry condition) (solid line, gray), and the remaining activity of each sample was measured at time points of 0, 1, 2, 4, and 8 weeks as described in Materials and Methods. All measured activities were normalized to the sample before the aging test.

Six CAH homologs in this study were evaluated with respect to thermostability in conditions important to the intended application of pool remediation (Fig. 7). Thermostability is relevant to the process of preparing CAH proteins in a stable dry granule, which would be used for storage, transport, as well as to deliver the catalyst in a convenient and practical form for swimming pool remediation. Granules were prepared and tested as described in the Materials and Methods section. Each CAH enzyme's performance at condition (1) room temperature was considered to be 100% and the performance at other conditions was normalized to this value for the plot. Under these conditions, a granule of CAH-PR could maintain >70% activity at 50°C or 40°C with humidity for 8 weeks (Fig. 8). The *k*_cat_ and *K*_m_ values of CAH-PR and CAH-BD were similar to each other at ∼10/s and 103–115 μM respectively, which were determined using the method described in a previous study (Seffernick et al., 2012). The kinetics parameters and retained activities for the two enzymes at high temperature and in storage condition are summarized and shown in Table 1.

**Table 1.** Kinetic Statistics and Thermostability of CAH-PR (*Pseudolabrys* sp. Root1462) and CAH-BD (*Bradyrhizobium diazoefficiens*) Proteins

Enzyme name/organism	*k*_cat_ (s^–1^)	*K*_m_ (μM)	Percentage retained at 75°C, 30 min	Percentage retained after storage^a^
CAH-PR/*Pseudolabrys* sp. Root1462	10.1 ± 2.7	115 ± 36	93.9%	67.4%
CAH-BD/*Bradyrhizobium diazoefficiens*	10.6 ± 1.9	103 ± 29	18.3%	48.0%

^a^CAH granules were stored at 50°C + 65% humidity for 8 weeks.

### Oxidation of Biuret With Sodium Hypochlorite

While biuret is the predominant product of enzymatic hydrolysis of CYA in solution, the fate of biuret in the presence of excess active chlorine is also important to understand. In particular, we sought to quantify the extent to which biuret is converted to gaseous versus water-soluble byproducts that would remain in the pool. We applied several analytical techniques (high-performance liquid chromatography [HPLC], GC/MS, total nitrogen analysis, and NMR) to follow the enzymatic conversion of CYA to biuret, and then characterized the end-products of biuret oxidation with excess hypochlorite. An isotope-labeling study was also performed in order to determine the mechanism by which N—N bonds were formed under conditions relevant to swimming pools.

Treatment of ^13^C_3_/^15^N_3_-labeled CYA with CAH-PR was monitored over time by both ^13^C-NMR and ^15^N-NMR and confirmed that ^13^C/^15^N-biuret was the main product, as described previously (Seffernick et al., 2012) and shown in Figs S2a and b. Enzyme (6 μl of 0.25 mg/ml CAH-PR) was added to an NMR tube and ^13^C-NMR spectra were recorded periodically to monitor conversion to ^13^C_3_/^15^N_3_-biuret. Additional enzyme (14 μl of 10 mg/ml) was added at the 2 hr time point to increase the rate of conversion to ^13^C_3_/^15^N_3_-biuret, which was complete after 4 hr at 25°C as judged by the disappearance of the ^13^C triplet signal centered at 154.6 ppm corresponding to residual ^13^C_3_/^15^N_3_-CYA.

Sodium hypochlorite (∼1 molar equivalent relative to CYA) was added to the NMR tube containing ^13^C_3_/^15^N_3_-biuret and vortexed briefly, resulting in the formation of gas bubbles over the course of a few hours. A ^13^C NMR spectrum was obtained (nt = 2,000) after the initial evolution of gas had subsided and revealed a partial decrease in the intensity of the ^13^C_3_/^15^N_3_-biuret signal. Increased signals at 127.3 and 162.8 ppm were also noted, corresponding to ^13^CO_2_ and ^13^C-bicarbonate, respectively. After 2 hr, additional bleach was added (to ∼3 eq. in total) and additional ^13^C spectra were acquired after gas evolution ceased, including a 24 hr time point, which resulted in further decreases in the ^13^C_3_/^15^N_3_-biuret signal (Fig. S2B). Trace signals at 131.3 and 131.4 ppm (d) and 159.5 ppm (t) and 159.1 ppm (t) were noted, possibly cyanate and chlorinated biuret or other side products. A bleach control did not contain any observable signals in the ^13^C NMR spectrum.

### ^15^N-NMR Study of CYA Hydrolysis and Biuret Chlorination

The experiment described previously was then repeated, this time followed by ^15^N-NMR. Prior to addition of CAH-PR, a signal corresponding to ^15^N_3_/^13^C_3_-CYA was observed at 135.5 ppm (Fig. S2C), appearing as a triplet. The ^13^C–^15^N coupling constant (*J*-value) was ∼18 Hz. The absence of a large N—H coupling was a function of the enol tautomer of CYA predominating in solution.

Addition of CAH-PR (6 μl of 0.25 mg/ml) resulted in slow conversion to ^13^C_3_/^15^N_3_-biuret, as evidenced by the loss of a signal (dd appearing as a triplet) centered at 135.5 ppm and the appearance of signals attributed to ^13^C_3_/^15^N_3_-biuret at 121.8/119.9 (dt) (imino ^15^N) and a ddd at 85.1 (amino ^15^N) (Figs S2D and E). Coupling constants of 19 Hz (^13^C–^15^N) and 90 Hz (^1^H–^15^N) were observed, along with smaller couplings for longer range ^1^H–^15^N interactions.

After CYA was completely hydrolyzed, an equivalent of sodium hypochlorite (NaClO) solution was added to the NMR tube containing ^13^C_3_/^15^N_3_-biuret and vortexed briefly. The ^15^N NMR spectrum was obtained (nt = 4,096) after the initial evolution of gas had subsided. Following this treatment, the ^13^C_3_/^15^N_3_-biuret signals were observed to diminish and a new signal at 310 ppm was observed, attributed to nitrogen gas (N_2_). Additional bleach (∼3 eq. in total) was added, followed by overnight NMR acquisition, and resulted in loss of observable biuret signals. Other than a residual N_2_ signal at 310 ppm, no other signals were observed. Analysis of the headspace in the NMR tube by GC/MS confirmed the presence of ^15^N_2_ and ^13^CO_2_ gas following enzymatic hydrolysis and bleach treatment of ^15^N_3_/^13^C_3_-CYA.

**Fig. 9.**
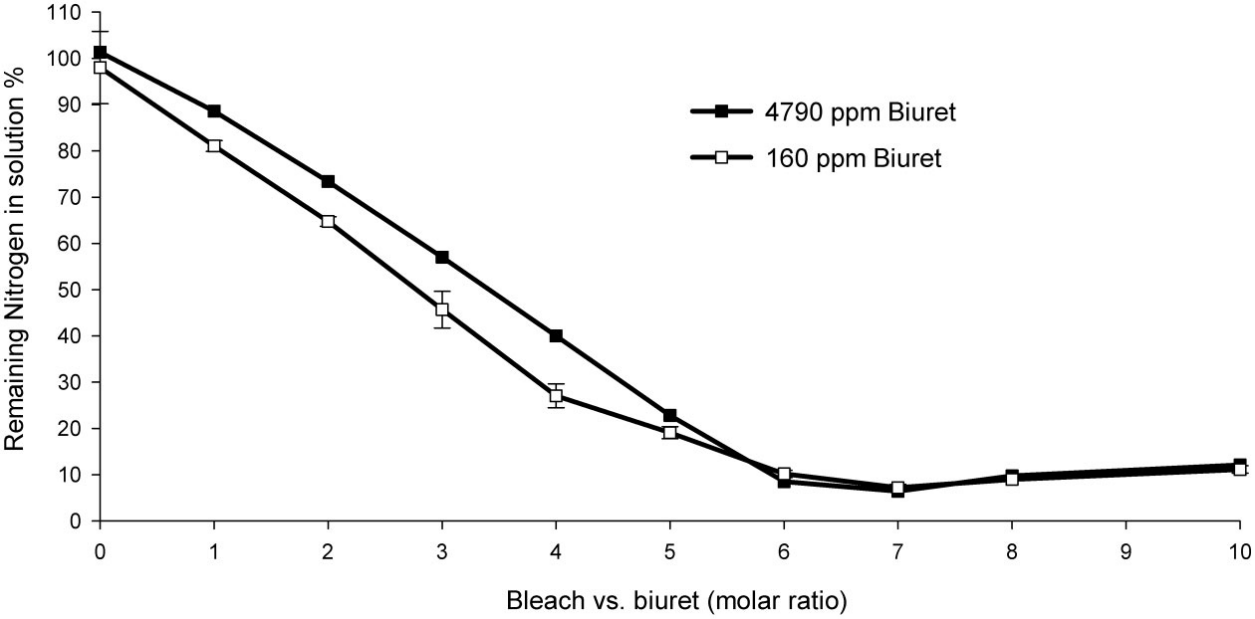
Titration of 0–10 molar equivalents of bleach (NaClO) against high (4790 ppm) and low (160 ppm) concentrations of biuret. High (4790 ppm, black) and low (160 ppm, gray) concentrations of biuret were titrated by bleach (NaClO) with 0, 1, 2, 3, 4, 5, 6, 7, 8, 9, and 10 molar equivalents and incubated at room temperature for 24 hr, followed by analysis of the remaining nitrogen in solution. The amount of remaining nitrogen in each sample was normalized to the sample without bleach.

In a separate study, HPLC was used to determine the nature and amount of soluble byproducts following bleach treatment (0–10 molar equivalents) of an unlabeled biuret standard (generated from enzymatic hydrolysis of CYA by CAH-PR). The remaining nitrogen in the solution was converted to nitrate (but not nitrite) as determined by nitrate and nitrite test strips (Hach, Loveland, CO, USA) and HPLC (data not shown). Total nitrogen analysis was applied to characterize the extent to which nitrogen was removed from the system upon bleach treatment. Two concentrations of unlabeled biuret (high and low, or 4,790 and 160 ppm, respectively) were titrated with 0–10 molar equivalents of bleach (Clorox, Oakland, CA) before analyzing the remaining nitrogen in solution. Briefly, 100 ml of 4,790 ppm biuret was prepared by incubating 6,000 ppm of CYA with 5 mg/l of CAH-PR at room temperature overnight. Complete hydrolysis of CYA to biuret was confirmed by melamine cyanurate precipitation assay. Biuret solutions (10 ml of either 4,790 or 160 ppm) were mixed with bleach (NaClO) at 0, 1, 2, 3, 4, 5, 6, 7, 8, and 10 molar equivalents in test tubes and incubated at room temperature for 24 hr. For the high-concentration samples, the remaining amount of nitrogen was determined using an ECS 4010 CHNSO elemental analyzer (Costech, Valencia, CA) by applying 20 μl of each sample with BBOT (2,5-bis [5-*tert*-butyl-benzoxazol-2-yl] thiophene) as the nitrogen standard. For the lower concentration (160 ppm biuret) samples, 500 μl of 1 M Na_2_SO_3_ was added to each sample to neutralize the remaining bleach before concentrating each 10 mL sample to ∼300 μl by SpeedVac (Labconco, Kansas City, MO) at 40°C. The amount of nitrogen in biuret without bleach was set at 100% and all the other samples were normalized to this value. As shown in Fig. 9, only ∼10% of the initial nitrogen remained in solution after more than six equivalents of bleach had been added, indicating ∼90% of the nitrogen initially present was converted to gaseous byproducts.

### Mechanistic Investigation With ^15^N-Urea and ^15^N-Biuret Oxidation by Hypochlorite

The mechanism by which N_2_ was formed upon oxidation of both urea and biuret with hypochlorite was studied using a 1:1 mixture of ^14^N- and ^15^N-labeled standards, followed by GC/MS analysis of the resulting nitrogen isotopomers present in both N_2_ and N_2_O.

A typical GC/MS chromatogram is shown in Fig. S3A with N_2_ eluting at 1.84 min, CO_2_ at 2.62 min, and traces of N_2_O at 2.88 min. Reactions were conducted at concentrations relevant to recreational pools (∼1 mM) and at three different pH values (∼5, 7, and 10) using an excess (>10-fold) of sodium hypochlorite solution (commercial Clorox bleach). The ratio of ions at *m*/*z* 29 and *m*/*z* 30, corresponding to ^14^N–^15^N and ^15^N–^15^N dinitrogen isotopomers, was determined after correction for background nitrogen from the air (99.63% ^14^N–^14^N). The observed *m*/*z* 29 versus *m*/*z* 30 ion ratios for both urea and biuret at the three pH values tested, and the corresponding fraction of intramolecular reaction for each condition, are shown in Table 2.

**Table 2.** Observed *m*/*z* 29 Versus *m*/*z* 30 Ion Ratios for Both Urea and Biuret at Three pH Values Tested and the Corresponding Percentage of Intramolecular Reaction Leading to N_2_ Formation

Condition	Ratio of *m*/*z* 29/30	Percentage of intramolecular N_2_ formation
1 mM urea–pH 5	1.78	5.8
1 mM urea–pH 7	1.76	6.3
1 mM urea–pH 10	0.93	36.6
1 mM biuret–pH 5	1.99	0.3
1 mM biuret–pH 7	1.97	1.1
1 mM biuret–pH 10	1.44	24.6

Similarly, the ratio of ions at *m*/*z* 45 and 46 was estimated for the trace amounts of N_2_O detected and was ∼2 in all samples observed, including experiments run at higher concentrations (data not shown).

## Discussion

The data reported in this work show that all CAH enzymes studied to date are quite sensitive to inactivation by hypochlorite. Further work could be done to quantify the specific rate and affinity constants for CAH inactivation, but given the results shown here the most practical method to relieve inactivation during enzymatic CYA remediation is *in situ* chemical reduction of active hypochlorite, or other oxidative species, prior to addition of CAH. Clearly, many other configurations of reduction or removal of oxidative species could be considered, but the method shown here is practical for swimming pool applications.

Among the six CAH homologs tested in this study, CAH-PR and CAH-BD were found to be the most robust with respect to the application and production process described for swimming pool remediation and maintenance in this study. The absolute enzyme requirements will vary depending on each specific application, as well as manufacturing specifics, including production yield, temperature of protein recovery, purification, granulation, storage, and in-pool application. For the specific conditions used in our study, CAH-PR had improved storage stability relative to CAH-BD (Figs 7 and 8 and Table 1), but both enzymes were markedly more suitable for CYA remediation than the other CAH candidates tested.

Structure superposition of CAH-PR and CAH-BD homology models with *Pseudomonas* sp. ADP (PDB:4BVQ) (Peat et al., 2013), *M. thermoacetica* CAH (PDB: 6BUM) (Shi et al., 2019), and *A. citrulli* (122227) TrzD (PDB: 5T13) (Bera et al., 2017) by molecular operating environment (Chemical Computing Group ULC, Montreal, QC, Canada) showed that these enzymes had very similar folding and overall structures (Fig. S4, generated by PyMOL [Yuan et al., 2016]). The catalytic residues of CAH are typically defined by three groups of arginine–lysine–serine triads from three domains (Shi et al., 2019). Sequence alignment and structure superposition of CAH-PR and *M. thermoacetica* (6BUM) showed that these catalytic residues and the consensus sequence at the C-terminus were identical and at the same position, indicating that the observed thermostability was not due to structural variation within the active sites or the consensus sequence. We hypothesize that the enhanced thermostability of CAH-PR might contribute to the quaternary stability of this protein, which stabilizes and maintains overall protein folding at high temperature, therefore preventing protein denaturation. Further investigation with structure-guided mutagenesis might be used to illustrate the mechanism in further detail.

Monitoring the free chlorine concentration before and after adding the reductant is a key step for successful enzyme treatment. Ideally, any free hypochlorite should be at undetectable levels after adding the reductant. The chemistry involved with the chlorination of water is somewhat complex. The equilibrium of hypochlorous acid, chlorine, and hypochlorite ion in water is a function of both pH and temperature (Fig. S1[1]). Collectively, these oxidizing chemical species are formed when bleach (sodium hypochlorite) is dissolved in water, and are referred to as “free residual chlorine” or “free available chlorine,” whereas chlorinated species resulting from reaction with ammonia and/or organic nitrogen compounds are referred to as “combined chlorine.” The term “total residual chlorine” refers to the sum of “free chlorine” and “combined chlorine.” Colorimetric analytical methods are conveniently used to determine chlorine levels in water. In general, the reagent used in most colorimetric methods is DPD, which can be used to measure both free and total chlorine in water. In our case, we adapted a commercial DPD assay to a quantitative 96-well plate format.

The CYA decomposition rate under typical swimming pool conditions is reported to be very low, which is why CYA is used as a hypochlorite stabilizer (Wojtowicz, 2001). CAHs initially generate carboxybiuret by amide-bond hydrolysis of CYA, which then spontaneously hydrolyzes to biuret and CO_2_ (Aukema et al., 2020). Whereas CO_2_ partitions to the air, depending on the water pH and temperature, biuret is difficult to remove from pool water at the desired pH values of 7.0–7.5. Given this issue, we studied several chemical and/or physical approaches to remove the biuret resulting from enzymatic CYA hydrolysis. The p*K*_a_ of the biuret amide-NH_2_ protons is ∼10.2, making it possible in principle to use anion exchange resins for biuret removal, but in practice this approach would require a large amount of resin and would drive up maintenance costs. Other absorbents for biuret removal, such as activated carbon and AlCl_3_, are likely impractical for large pools and require additional solids handling steps. Using potassium peroxymonosulfate as a bleach substitute also failed to remove biuret from pool water in our hands (data not shown), although it is commonly applied in the pool industry to shock swimming pools.

In contrast to CYA, we found that biuret was readily oxidized upon addition of excess hypochlorite. Further investigations of the products resulting from this reaction were conducted in simulated pool water by NMR and total nitrogen analysis (Fig. S2 and Fig. 9). The oxidation of urea, biuret, and other nitrogenous substances with hypochlorite and hypobromite was studied independently by both Fenton and Foster in the 19th century (Fenton, 1878; Foster, 1879). They concluded that oxidation under alkaline conditions produced both gaseous (N_2_, N_2_O, and CO_2_) and soluble (NO_2_, NO_3_, and OCN) products. With hypochlorite, urea and biuret released only half and a third of their total nitrogen, respectively. The remainder was thought by Fenton to be a cyanate salt. Hypobromite, a stronger oxidant than hypochlorite, was observed to release most, but not all, of the nitrogen from urea and biuret as nitrogen gas.

Chlorination of both urea and biuret under high-pH conditions (>pH 12) with stoichiometric amounts of hypochlorite affords a route to hydrazine, an alternative to the Raschig process, whereby ammonia is converted to hydrazine via chloramines (Schirmann & Bourdauducq, 2001). An intramolecular mechanism for N—N bond formation from both urea and biuret was proposed in mechanistic studies of this reaction. This reaction could proceed by either the Hofmann- or Favorskii-type mechanisms, as discussed in detail by Francis and Lim (2007).

Subsequent studies of urea chlorination at pH values and concentrations more relevant to recreational pool applications suggest a complex mechanism as proposed by Blatchley and Cheng (2010). In this case, dinitrogen is formed exclusively through an intermolecular process, with chloramines acting as the key intermediates. Blatchley proposed that in contrast to chlorination of ammonia, chlorination of urea results in direct formation of di- and trichloramine—compounds known to be responsible for the strong chlorine odor of pools where significant amounts of organic nitrogen are present.

In the current investigation, it was confirmed that similar to urea, the final products following oxidation of dilute biuret solutions with 6–10 molar equivalents of hypochlorite were CO_2_, N_2_, traces of N_2_O, and nitrate as confirmed by HPLC, GC/MS, and NMR. Both ^13^C- and ^15^N-NMR were used to follow the initial enzymatic conversion of ^13^C/^15^N-CYA to ^13^C/^15^N-biuret, and then the subsequent oxidation of ^13^C/^15^N-biuret with sodium hypochlorite (Fig. S2). Analysis of reaction mixtures by HPLC indicated that some nitrogen was converted to nitrate and remained in solution under these conditions, amounting to ∼10% of the total nitrogen initially present (Fig. 9). As shown in the hypothetical mechanism for N chlorination of biuret, 4.5 molar equivalents of hypochlorite were required to drive the oxidation state of nitrogen from −3 to 0 (N_2_) (Fig. S1[7]), while more hypochlorite was required to drive it to +5 (HNO_3_) (Fig. S1[8]).

**Scheme 1.**
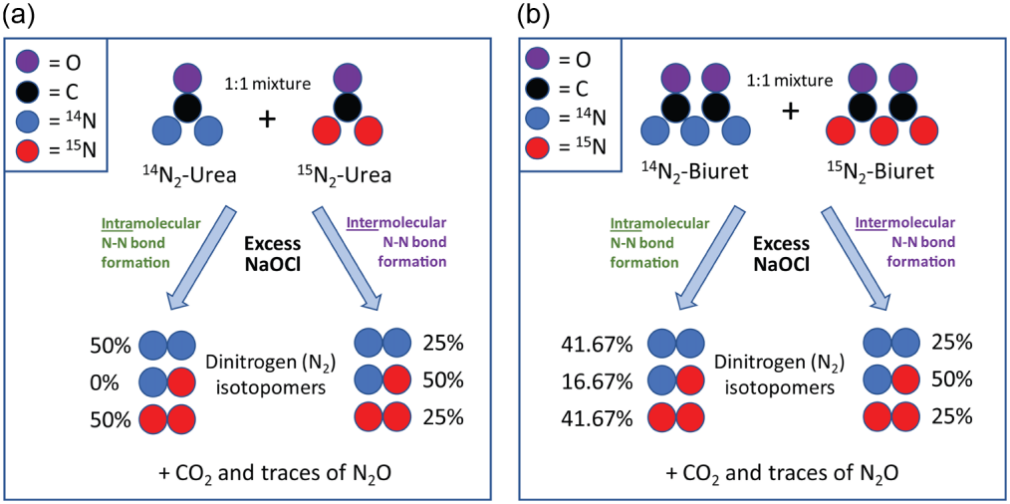
Expected ^14^N/^15^N isotope ratios for dinitrogen (N_2_) produced upon hypochlorite oxidation of a 1:1 mix of (a) ^14^N- and ^15^N-urea and (b) ^14^N- and ^15^N-biuret. In both cases, the intermolecular mechanism produces a 1:2:1 mixture of ions at *m*/*z* 28, 29, and 30. The intramolecular mechanism would produce only *m*/*z* 28 and 30 in the case of urea. For biuret, the intramolecular route would only account for two of the three nitrogen atoms, giving a ratio of 5:2:5 for ions 28, 29, and 30.

An isotope labeling strategy was applied to differentiate between an intramolecular mechanism for initial N—N bond formation upon hypochlorite oxidation of both urea and biuret, and an intermolecular mechanism whereby dinitrogen is formed after amide-bond cleavage through chloramine intermediates. Treatment of a 1:1 mixture of unlabeled urea (^14^N, 99.63%) and ^15^N_2_-labeled urea (^15^N, 99%+) with excess sodium hypochlorite was conducted under dilute (1 mM total urea) concentrations at mildly acidic, neutral, and basic conditions (pH values of 5, 7, and 10). The ratios of ions *m*/*z* 29 (^14^N–^15^N dinitrogen) and *m*/*z* 30 (^15^N–^15^N dinitrogen) were determined and compared with the theoretical outcomes for each mechanism, as depicted in Scheme 1. A typical GC/MS trace is shown in Fig. S3A, with N_2_ eluting at 1.84 min, CO_2_ at 2.62 min, and N_2_O at 2.88 min. The ratio of ions at *m*/*z* 29 to *m*/*z* 30 after oxidation of urea (Fig. S3B) and biuret (Fig. S3C) was determined and used to calculate the ratio of intra- to intermolecular reaction mechanisms. The results indicated that the intermolecular reaction mechanism predominates at all pH values for both urea and biuret, but also revealed that at basic pH the intramolecular mechanism accounts for 36% of the N_2_ produced from urea and ∼25% of the N_2_ derived from biuret (Table 2). Under conditions relevant to swimming pools, the results of this study support that hypochlorite oxidation of biuret produces mostly gaseous by-products in a similar manner to urea (Blatchley & Cheng, 2010) (Fig. S1[6–8]).

Overall, we expressed and characterized two previously unknown CAH enzymes from *B. diazoefficiens* and *Pseudolabrys sp.* Root1462 and demonstrated the latter to have properties rendering it suitable for CYA removal from recreational swimming pools. We also developed a process involving chemical reduction to remove residual hypochlorite, subsequent enzymatic hydrolysis of CYA to biuret, and finally oxidation of the resulting biuret to primarily gaseous by-products. The mechanism of biuret oxidation was further investigated and found to be similar to the oxidation of urea under conditions relevant to swimming pools. The approach described in this work offers a sustainable means to manage chlorine levels, prevent CYA accumulation, and reduce water consumption in the recreational swimming pool industry.

## Supplementary Material

kuab084_Supplemental_File
