# Supplementary material for: A procedure for removal of cyanuric acid in swimming pools using a cell-free thermostable cyanuric acid hydrolase
Source: J Ind Microbiol Biotechnol. 2021 Nov 12;49(2):kuab084. doi: 10.1093/jimb/kuab084 (PMC9118981; doi:10.1093/jimb/kuab084)
Supplement: kuab084_Supplemental_File [file kuab084_Supplemental_File.docx]

**A Procedure for Removal of Cyanuric Acid in Swimming Pools Using a Cell-Free Thermostable Cyanuric Acid Hydrolase**

**Feng Guo^1^, Joseph C. McAuliffe^1^, Cristina Bongiorni^1^, Jacob A. Latone^1^, Mike J. Pepsin^1^, Marina S. Chow^1^, Raj S. Dhaliwal^1^, Katherine M. Hoffmann^1^, Bill T. Brazil^1^, Meng H. Heng^1^, Serina L. Robinson^3^, Lawrence P. Wackett^2^, and Gregory M. Whited^1^ ***

**1 Nutrition & Biosciences, International Flavors and Fragrances Inc., Palo Alto, California, USA**

**2 Department of Biochemistry, Molecular Biology and Biophysics, University of Minnesota, Minneapolis, Minnesota, USA**

**3 Department of Environmental Microbiology, Eawag: Swiss Federal Institute for Aquatic Science and Technology, Überlandstrasse 133, CH-8600, Dübendorf, Switzerland**

*** Correspondence: Gregg.Whited@iff.com**

**Supplemental figures:**

**Fig. S1** **Chemistry reactions and mechanism related to CYA function, hydrolysis of CYA using CAH, and biuret chlorination.**

(1) Dissociation of hypochlorous acid in aqueous solution; (2) Chlorination of CYA by hypochlorite to form trichloro-CYA as a chlorine stabilizer in swimming pool. The reaction is reversable, therefore to release and maintain free chlorine for sanitation; (3) Hydrolysis of CYA using CAH with biuret and CO_2_ as by products; (4) Neutralization of hypochlorite by sodium sulfite; (5) Neutralization of hypochlorite by sodium thiosulfate; (6) Hypothetical reaction mechanism for nitrogen chlorination of biuret by hypochlorite at initial step with intermediate product; (7) Hypothetical reaction mechanism of biuret chlorination to form carbon dioxide and nitrogen gas as final products; (8) Hypothetical reaction mechanism of biuret chlorination to form carbon dioxide and nitrate as final products.

**Fig. S2 Tracking the generation of biuret from ^13^C/^15^N-labelled CYA by CAH-PR and followed by bleach treatment in NMR.**Fig. S2A: ^13^C NMR spectrum with partial digestion of ^13^C/^15^N-labelled CYA by CAH-PR. CYA showed a triplet centered at 154.3 ppm, biuret peak was observed as a doublet of doublets (dd) centered at 160.09 ppm (signals at 159.93, 160.08, 160.10, 161.25 ppm), while a singlet attributed to bicarbonate was observed at 162.86 ppm.

Fig. S2B: ^13^C NMR spectrum of ^13^C/^15^N-labelled biuret mixed with 3 molar equivalents of bleach (NaClO). The signal of biuret (dd at 160.09 ppm) was decreased, while increased signals at 127.32 ppm and 162.8ppm corresponding to ^13^CO_2_ and ^13^C-bicarbonate were observed.

Fig. S2C: ^15^N NMR spectrum of ^13^C/^15^N-labelled CYA before addition of CAH-PR. A signal corresponding to ^15^N/^13^C-CYA was observed at 135.5 ppm.

Fig. S2D: ^15^N NMR spectrum with partial hydrolysis of ^13^C/^15^N-labelled CYA by CAH-PR. Addition of CAH-PR resulted in the appearance of signals attributed to ^15^N/^13^C-biuret at 121.76/119.95 (dt) (imino ^15^N) and a ddd at 85.05 (amino ^15^N).

Fig. S2E: ^15^N NMR spectrum with complete digestion of ^13^C/^15^N-labelled CYA by CAH-PR.

Upon complete digestion of CYA to biuret, signal corresponding to ^15^N/^13^C-CYA at 135.5 ppm disappeared and signals attributed to ^15^N/^13^C-biuret at 121.76/119.95 (dt) (imino ^15^N) and a ddd at 85.05 (amino ^15^N) remained on the spectrum.

**Fig. S3 GC/MS chromatogram of dinitrogen formation upon oxidation of urea and biuret.**

Fig. S3A: A typical GC/MS chromatogram of the gaseous products from bleach treatment (>10-fold) using biuret standard under conditions relevant to swimming pools. As a result, nitrogen elutes at 1.84 min, carbon dioxide at 2.62 min and nitrous oxide at 2.88 min, respectively.

Fig. S3B: GC/MS chromatograms of the gaseous products from bleach treatment (>10-fold) using 1:1 mixture of unlabeled and ^15^N-labeled urea standard at pH 5, 7 and 10. At all three pH conditions, the ratio of ions at m/z 29 (blue) and m/z 30 (red), corresponding to ^14^N-^15^N and ^15^N-^15^N dinitrogen isotopomers from the bleach treatment of urea, was determined after correction for background nitrogen from the air (99.63% ^14^N-^14^N).

Fig. S3C: GC/MS chromatograms of the gaseous products from bleach treatment (>10-fold) using 1:1 mixture of unlabeled and ^15^N-labeled biuret standard at pH 5, 7 and 10. At all three pH conditions, the ratio of ions at m/z 29 (blue) and m/z 30 (green), corresponding to ^14^N-^15^N and ^15^N-^15^N dinitrogen isotopomers from the bleach treatment of biuret, was determined after correction for background nitrogen from the air (99.63% ^14^N-^14^N).

**Fig. S4 Structure superposition of CAH-PR/CAH-BD and CAH homologues from three bacterial genera.** Homology models of CAH-PR and CAH-BD were built and superposed in MOE using published CAH structures of *Pseudomonas sp.* ADP (PDB: 4BVQ), *M. thermoacetica* CAH (PDB: 6BUM) and *Acidovorax citrulli* (122227) TrzD (PDB: 5T13) as template, and the figure was generated in PyMOL. Homology models of CAH-PR and CAH-BD were shown as black and cyan ribbons, respectively; while 4BVQ, 5T13 and 6BUM were shown as ribbons colored in magenta, orange and green respectively. The arginine-lysine-serine catalytic triads from CAH-PR were presented as surface and colored in red, yellow and blue for K40-R52-S80, K156-R188-S226 and K294-R323-S342, respectively. CAH-PR and CAH-BD share an average of 49.4% and 57.0% sequence identity with three CAH homologs respectively. The overall RMSD values between CAH-PR/CAH-BD homology models and these three structures are 1.213 Å and 1.022 Å, respectively.
